# Use of unconventional antithyroid therapy in patients with thiamazole agranulocytosis in the context of the COVID-19 pandemic

**DOI:** 10.20945/2359-3997000000469

**Published:** 2022-04-28

Arch Endocrinol Metab. 2022;66(1):132-3

Where you read:

**Julio Alvarez Gamero** ^1^

https://orcid.org/0000-0002-6861-5699

**Victor García Ruiz** ^2^

https://orcid.org/0000-0002-6846-7630

**Jose Paz Ibarra** ^3^

https://orcid.org/0000-0002-2851-3727

Should read:

**Julio César Alvarez-Gamero** ^1^

https://orcid.org/0000-0002-6861-5699

**Victor Raúl García-Ruiz** ^2^

https://orcid.org/0000-0002-6846-7630

**Jose Luis Paz-Ibarra** ^3^

https://orcid.org/0000-0002-2851-3727

